# The Consolidated Framework for Implementation Research (CFIR): a useful theoretical framework for guiding and evaluating a guideline implementation process in a hospital-based nursing practice

**DOI:** 10.1186/s12912-015-0088-4

**Published:** 2015-08-12

**Authors:** Helga E. Breimaier, Birgit Heckemann, Ruud J. G. Halfens, Christa Lohrmann

**Affiliations:** Institute of Nursing Science, Medical University of Graz, Billrothgasse 6, 8010 Graz, Austria; Department of Health Services Research, CAPHRI, Maastricht University, Duboisdomein 30, 6229 GT Maastricht, The Netherlands

**Keywords:** Consolidated Framework of Implementation Research (CFIR), Evaluation, Guideline implementation, Nursing

## Abstract

**Background:**

Implementing clinical practice guidelines (CPGs) in healthcare settings is a complex intervention involving both independent and interdependent components. Although the *Consolidated Framework for Implementation Research* (CFIR) has never been evaluated in a practical context, it appeared to be a suitable theoretical framework to guide an implementation process. The aim of this study was to evaluate the comprehensiveness, applicability and usefulness of the CFIR in the implementation of a fall-prevention CPG in nursing practice to improve patient care in an Austrian university teaching hospital setting.

**Methods:**

The evaluation of the CFIR was based on (1) team-meeting minutes, (2) the main investigator’s research diary, containing a record of a before-and-after, mixed-methods study design embedded in a participatory action research (PAR) approach for guideline implementation, and (3) an analysis of qualitative and quantitative data collected from graduate and assistant nurses in two Austrian university teaching hospital departments. The CFIR was used to organise data per and across time point(s) and assess their influence on the implementation process, resulting in implementation and service outcomes.

**Results:**

Overall, the CFIR could be demonstrated to be a comprehensive framework for the implementation of a guideline into a hospital-based nursing practice. However, the CFIR did not account for some crucial factors during the planning phase of an implementation process, such as consideration of stakeholder aims and wishes/needs when implementing an innovation, pre-established measures related to the intended innovation and pre-established strategies for implementing an innovation. For the CFIR constructs *reflecting & evaluating* and *engaging,* a more specific definition is recommended. The framework and its supplements could easily be used by researchers, and their scope was appropriate for the complexity of a prospective CPG-implementation project. The *CFIR* facilitated qualitative data analysis and provided a structure that allowed project results to be organised and viewed in a broader context to explain the main findings.

**Conclusions:**

The CFIR was a valuable and helpful framework for (1) the assessment of the baseline, process and final state of the implementation process and influential factors, (2) the content analysis of qualitative data collected throughout the implementation process, and (3) explaining the main findings.

**Electronic supplementary material:**

The online version of this article (doi:10.1186/s12912-015-0088-4) contains supplementary material, which is available to authorized users.

## Background

Several implementation theories are available for use when translating research-based knowledge, such as evidence-based clinical practice guidelines (CPGs), into hospital-based nursing practice. These implementation theories refer to influential factors, but identical or similar factors are often referred to with different names in different theories. Furthermore, implementation theories are often not exhaustive with regard to these factors. Therefore, Damschroder et al. combined 19 published implementation theories into the Consolidated Framework for Implementation Research (CFIR). The CFIR includes five major domains (*intervention characteristics, outer setting, inner setting, characteristics of individuals* and *process*) with 39 underlying constructs and sub-constructs that can potentially influence efforts to change the practice [1]. Each (sub-) construct is defined; for example, *tension for change* is defined as “the degree to which stakeholders perceive the current situation as intolerable or needing change” ([1], p. 8). The constructs can be used as implementation and evaluation criteria in three different ways: they may (1) raise awareness for potential influential factors, (2) facilitate the analysis of pivotal processes and outcomes and (3) help organise all findings of an implementation process to explain the outcomes (i.e., to understand what worked where and why) [1].

According to Grol et al., the CFIR can be considered an explanatory framework. To complement its use, a ‘process’ or ‘action theory’ such as a participatory action research (PAR) approach is needed [2]. CFIR’s theory-based constructs and mechanisms can be used to help explain whether an implementation may or may not succeed. Furthermore, it can be used to identify potential barriers and facilitators if used before or during an implementation. This, in turn, helps guide the selection of strategies to overcome or affect these influential factors [3]. The PAR approach, with its cyclical process, may aid the identification of each relevant step in a CPG implementation process.

To be truly useful, the CFIR must be a comprehensive, applicable and helpful framework that meets the above-mentioned criteria listed by Damschroder et al. [1]. Since its publication in 2009, the CFIR has been applied in a number of studies to either explain or describe research findings, in order to identify matters of interest or evaluate the framework itself. Sorensen and Kosten [4] applied the framework to multiple articles appearing in one journal issue and found that the model could be useful to systematically describe the implementation research findings in a wide variety of clinical areas in the field of addictions. Damschroder and Lowery applied the CFIR as an interview guide and for analysis purposes in order to describe factors that explained variations in the implementation success of a programme that had been run in the Veteran’s Affairs medical facilities and illustrate how the CFIR could be applied to identify influential contextual constructs on the implementation process [5]. Damschroder and Lowery could thereby present specific examples to clarify the distinctions between a few constructs identified as being closely related during the CFIR’s application; for example *relative priority* versus *patient needs* and *resources; design quality and packaging* versus *access to knowledge and information* [5].

Damschroder and Hagedorn used the CFIR as an organisational framework to evaluate implementation theories used in substance abuse treatments. The authors concluded that the CFIR is a comprehensive practical taxonomy of constructs with the potential to influence implementation effectiveness. None of the evaluated implementation theories captured all constructs provided in the CFIR [3]. Hartzler et al. used the CFIR as a guide to explore publications for the transportability of contingency management in substance abuse treatment and to identify which of the CFIR domains had been treated in the examined literature [6]. The domains most often treated were *intervention characteristics* (59%) followed by *characteristics of individuals* (34%) and *inner setting* (32%). The examined literature focussed on *process* in 18% of the cases and *outer setting* in 8%. Powel et al. used the CFIR to examine ‘research (and real-world implementation efforts)’ with the expectation that this would give ‘some indication of how comprehensively strategies address important aspects of implementation’ ([7], pp. 194-195). The domains were addressed by implementation strategies in 45-100% of the included studies (*characteristics of individuals:* 100%, *inner setting:* 82%, *process:* 64%, *outer setting:* 55% and *characteristics of the intervention:* 45%). The authors appraised the CFIR as useful to gain a greater understanding of the overall designs of studies included in their systematic review and the intended targets of the implementation strategies [7].

In a post-hoc, deductive analysis of narrative accounts of innovation in health care services, Ilott et al. evaluated the utility of the CFIR. They found the framework to be both useful and user-friendly, capturing the complexity of implementation in health care practice within this context and across multiple sites. Additionally, it was deemed simple to apply due to its conceptual clarity and its wide coverage of the five domains [8]. No study was found that applied and evaluated the CFIR for its comprehensiveness, applicability and usefulness in an ongoing implementation project.

For this reason, the aim of the current study was to evaluate the comprehensiveness, applicability and usefulness of the CFIR itself. The evaluation of the CFIR’s comprehensiveness focussed on its constructs, while its usefulness and applicability were evaluated according to its application as a theoretical framework during the implementation process. The CFIR was applied as part of a fall-prevention CPG implementation project. The hospital-wide incidence of patient falls was used as a basis on which to implement the evidence-based guideline “Fall prevention for older and elderly persons in hospitals and chronic care facilities” [9] (subsequently referred to as the Falls CPG). The incidence of patient falls was considered to be high by the nursing management of the respective Austrian university teaching hospital. The goal of the implementation of the Falls CPG was to improve nursing practice in two of the hospital’s departments (Accident Surgery Department (ASD) & Ophthalmic Department (OD)). The project concentrated on the implementation and service outcomes [10]. The main focus of the previous study was to investigate the effectiveness of the implementation strategies used, and the resulting primary findings (service outcomes and resource use) were reported in Breimaier et al. [11].

## Methods

### Design

**Fig. 1:**
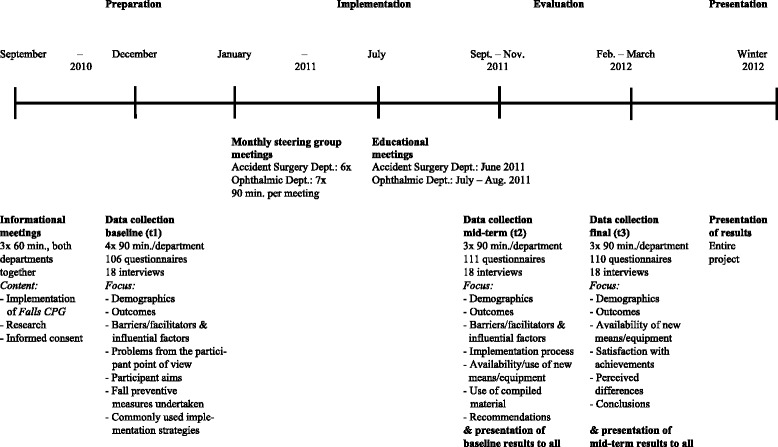
Course of the project

The CFIR’s comprehensiveness, applicability and usefulness were assessed through a post-hoc evaluation of how useful the framework actually was for guiding the implementation of the Falls CPG during the course of implementation. The comprehensiveness review focussed on the (1) presence or absence of necessary constructs as well as (2) the definitions of existing constructs. The applicability and usefulness review focussed on the CFIR (1) as a guide to develop assessment questions and as a framework to target influential factors, (2) as a template for content-analysis and (3) as a guide to interpret the main findings. The CFIR was applied within a before-and-after, mixed methods study design embedded in a participatory action research (PAR) approach for guideline implementation. An overview of the implementation process steps is given in Fig. 1.

### Procedure

The evaluation of the CFIR’s comprehensiveness, applicability and usefulness was based on data recorded during the implementation process. The main investigator’s research diary (RD), with consecutively entered notes, documented the course of the project. These included: decisions made; perceived facilitators, barriers and their solutions; topics and results of ad hoc meetings held on demand between HEB and head nurse or HEB, head nurse and ward managers from the two participating departments; observations and experiences made by HEB with regard to CFIR’s comprehensiveness and its application. Minutes of the steering group meetings (SGMMs) (January to July 2011) also document the course of the project. To assess the influential factors of the intended Falls CPG implementation project, as suggested by the CFIR [11], quantitative (questionnaire) and qualitative data (interviews, group discussions) were collected (baseline (t1), mid-term (t2), final (t3)).

Prior to the beginning of the implementation process, the CFIR was used to develop a list of questions, considering each CFIR domain and respective (sub-) constructs that was thought to be necessary and helpful for the assessment of the baseline of the project, could reveal influential factors during the course of the project and, finally, be used to evaluate the implementation process itself. This list of questions provided the basis for the applied questionnaires, respective semi-structured interview guides and reflective questions, which were asked during the steering group meetings. When analysing the qualitative data, the CFIR was used as a template for content analysis. Finally, the CFIR also was used as a guide to interpret the main findings. To do so, all findings obtained across all data sources per time point (interview, group discussions and questionnaire data, SGMMs, relevant RD entries) and across the three time points were organised under the respective CFIR domains, (sub-) constructs and its supplements (see Breimaier et al. [11]). The intention was (1) to reveal whether the qualitative and quantitative data, with regard to the same CFIR domain and (sub-) construct, supported each other or not, (2) find out how and which factors/elements influenced the implementation process and the main outcomes of the implementation project as suggested by the CFIR [1] and (3) discover changes that occurred over the course of the project. The strength and direction of the influence of the identified factors were assessed using the aggregated findings from all respective sources and marked with ‘+’ / ‘++’ (positive/very positive influence), ‘-’ / ‘--’ (negative/very negative influence) or ‘+/-’ (mixed influence). The main investigator’s thorough post-hoc reflections could be used as aids to combine relevant data for this assessment.

Details about the corresponding data collection tools and the respective data analysis methods are outlined in Additional file 1. The implementation and service results gained with respect to influential factors will be published elsewhere.

## Results

### CFIR comprehensiveness

#### Presence or absence of necessary constructs

The CFIR successfully covered a wide range of influential factors relevant for an implementation project, such as available resources, nursing personnel’s perception of the underlying problem (patient falls) and the intervention (Falls CPG) itself, communication and its channels, the culture of the organisation, co-operation within and between teams and leaders and the degree of receptiveness shown by the organisation with regard to implementation. However, during the preparation phase of the project, four constructs were added to the framework based on HEB’s reflection with regard to the implementation processes and her experience as a nurse (RD 23/07/2010): (1) stakeholders’ aims—it was assumed that the nursing personnel had their own ideas about what they wanted to achieve with regard to the Falls CPG implementation, (2) stakeholders’ wishes/needs when implementing an innovation like the Falls CPG—information about what should be considered in the ongoing implementation process was collected, (3) pre-established strategies for the implementation of an innovation—information about what was known and common and what may fit or not was collected and (4) pre-established (fall preventive) measures related to the intended innovation—it was assumed that fall prevention is within nursing personnel’s scope of responsibility for which measures exist.

#### Definitions of existing constructs

The underlying *reflecting & evaluating* construct (*process* domain) was defined very broadly as “quantitative and qualitative feedback about the progress and quality of implementation…” [1]. In the ongoing process followed during steering group meetings, the outcomes of the baseline assessment formed the basis for further action, which was repeatedly evaluated in each steering group meeting (see Fig. 1). To evaluate the proceedings from the nursing personnel’s perspective during the mid-term and final data collection periods (RD 21 & 24/05/2010, RD 20/07/2011, RD 23/02/2012), the definition of this construct was specified as: stakeholders’ conclusions about their satisfaction and contentedness with the project; project progress and achievements; the invested time and effort on behalf of stakeholders; the perceived change and impact; and stakeholders’ learning curves, as well as perceived barriers and facilitators. Additionally, stakeholder recommendations for further CPG implementation projects and assessments of the sustainability of the implemented Fall CPG were requested.

The underlying *engaging* construct (*process* domain) only considered persons involved in the design and realisation of the implementation process (for example, opinion leaders or external change agents), but not members of staff who applied the CPGs after implementation (i.e., the ‘frontline’ stakeholders). However, since this group’s degree of engagement was crucial to the project’s success, it was determined that this group would also be included as an additional sub-construct (RD 24/05/2010, RD 20/07/2011).

### CFIR applicability and usefulness

#### The CFIR as a guide to developing assessment questions and as a framework for revealing influential factors on the implementation process

The CFIR was simple to apply and supportive. It was helpful for the development of assessment questions for the Falls CPG implementation project. Moreover, questions that guided reflections on and evaluations of the progress within the steering group meetings were included as an integral part of each meeting (ASD & OD SGMMs).

**Fig. 2:**
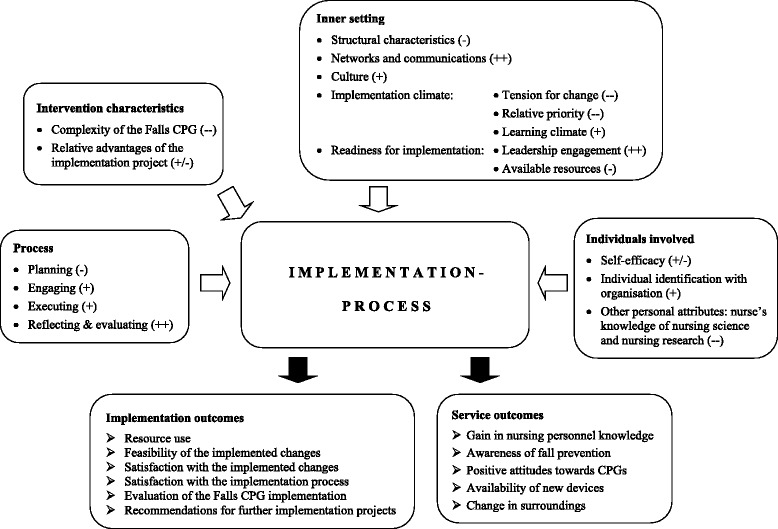
Overview of main influential factors on implementation process ordered by CFIR domain and (sub-) constructs, and achieved implementation and service outcomes. Legend: + positive / ++ very positive influence; - negative / -- very negative influence; +/- mixed influence

Several factors that influenced the Falls CPG implementation project were revealed—both as barriers and facilitators. Factors that mainly influenced the Falls CPG implementation emerged from four out of the five CFIR domains: *characteristics of the intervention, inner setting, characteristics of the individuals* and *process*. The respective (sub-) constructs that mainly influenced the implementation process are illustrated in Fig. 2 together with a description of the outcomes achieved. Constructs of the *outer setting* were not so highly influential during this implementation project and, therefore, are not included in the figure.

#### The CFIR as a template for content-analysis

In general, the task of assigning text passages from the interviews and group discussions to their respective CFIR construct or sub-construct was unproblematic. During the analysis of the first interviews (t1), however, the difference between *tension for change* and *relative priority* (domain *inner setting*, construct *implementation climate*) was revealed as not clear. An examination of the respective definition given by Damschroder et al. [1] prompted the decision that *tension for change* would refer to an individual person’s perspective and *relative priority*, to the organisation’s perspective (RD 15/12/2010, 05/01/2011). Furthermore, a close examination of the respective definition allowed us to distinguish between *adaptability* (domain *intervention characteristics*) and *compatibility* (domain *inner setting*) (RD 10/01/2011). Engagement or non-engagement, as expressed by interviewees (t2 & t3), could not clearly be assigned to either the *engaging* or to the *executing* construct (both constructs belong to the *process* domain). Based on a discussion between HEB and BH, a new sub-construct was created, *stakeholder involvement.* This sub-construct was finally assigned to the code *reflecting & evaluating* (domain *process*), because it reflected stakeholders’ own perspective about their engagement in the project (RD 13, 22, 24 & 27/01/2012). *Stakeholder involvement* was subsequently defined as the way in which stakeholders were incorporated into the project. During the analysis of the final interviews (t3), no further problems arose regarding content categorisation.

#### The CFIR as a guide to interpret the main findings

**Fig. 3:**
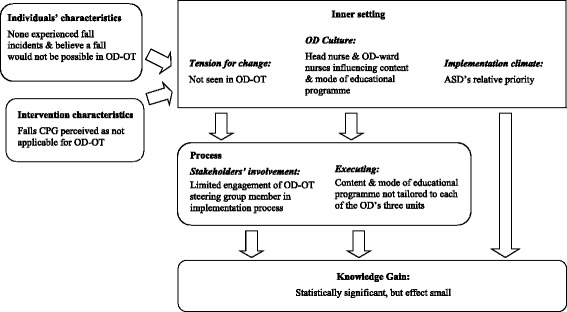
Limiting factors on nursing personnel’s knowledge gain on fall prevention. Legend: ASD = Accident Surgery Department, CPG = clinical practice guideline, OD = Ophthalmic Department, OT = operation theatre

Overall, and bearing in mind the information pooled under the CFIR’s domains and (sub-) constructs, the Falls CPG implementation project with its achieved outcomes could be considered successful, and the applied multifaceted and tailored implementation strategies were effective. The knowledge gain with regard to prevention of patient falls, which was one main outcome of the study, improved significantly (*p* = .001) between t1 and t3 [11]. However, the increase totalled 4.1 % and was, therefore, considered to be overall small. A closer look at factors that were presumed to limit the gain of knowledge in nursing personnel provided an explanation for this phenomenon. Constructs of *intervention* and *individuals characteristics* had an influence on the *tension for change* construct. This construct together with OD *culture* (*inner setting* domain) influenced the two *process* constructs*stakeholders’ involvement* and *executing*, respectively. We hypothesise that these two *process* constructs together with *implementation climate* (*inner setting* domain) negatively influenced nursing personnel’s knowledge gain. An overview is given in Fig. 3.

## Discussion

This study was the first to apply and, subsequently, evaluate the CFIR for its comprehensiveness, applicability and usefulness within a large-scale CPG-implementation project in acute nursing care. Herein, the CFIR guided the assessment of context and process, as well as the content-analysis of qualitative data. Moreover, it facilitated the organisation of influential factors and outcomes, which helped us explain the main results. According to Powell et al., the CFIR provides one of the most comprehensive overviews of the key theories and conceptual models that is a guiding force in implementation research and practice [7].

### CFIR comprehensiveness

#### Presence or absence of necessary constructs

Applying the framework and its five domains and 39 underlying (sub-) constructs in the Falls CPG implementation project helped all involved parties draw a comprehensive picture of the context of the two participating departments and the implementation process. Ilott et al. concluded from their examination of the terminology’s coherence that the framework offered a comprehensive structure and consistent terminology for scrutinising implementation *in situ* [8]. Yet, when applying the CFIR in this Fall CPG implementation project, it became apparent that the framework lacked important aspects for baseline assessment. Firstly, the framework did not take into account the fact that stakeholders, depending on their role within the organization, have disparate ideas, aims, wishes and requirements with regard to an innovation. These need to be considered and discussed openly when designing a change process, not only to win stakeholder acceptance and increase involvement, but also to facilitate the identification and management of barriers and facilitators. The constructs *stakeholders’ aims* and *stakeholders’ wishes/needs when implementing an innovation*, which were identified as being important in the present project, were lacking in the CFIR. The new constructs would enhance the *characteristics of individuals’* domain*.* According to its definition, individuals are, among other things, interested parties [1].

Secondly, the framework did not consider the fact that an innovation in healthcare is never introduced in a neutral environment, devoid of pre-existing work practices or change strategies. To make the right choice with regard to implementation strategies, it is essential to consider the existing conditions at the point an innovation is introduced (i.e., pre-existing structures and measures). It would make little sense to introduce pre-existing aspects that are already in line with recommendations of the guideline to be implemented. Such an introduction might strengthen the recommendations, but would waste energy and resources, both of which are limiting factors in the contemporary healthcare system, and could potentially create opposition among those involved. Those impacted by introduced changes must be addressed wherever they happen to be at that particular point in time ([12], p. 219). To augment the baseline assessment, an additional construct, labelled *pre-established measures related to the intended innovation*, could be included as part of the *implementation climate* construct of the *inner setting* domain.

Since the nursing work environment is characterized by constant change, it was assumed that nursing personnel had previous experience with alterations in processes and strategies. Including a setting’s *pre-established strategies for implementing an innovation* could also increase the practical value of the framework, because this would tell the implementers what might or might not be utilised in the implementation process and could provide tips for applicable implementation strategies. This additional construct could be included as a seventh construct to the *inner setting* domain. In this sense, the CFIR is in line with the recommendations of Ilott et al. [8]. They, however, suggested adding a sixth domain that would pertain to practical strategies in the CFIR. From their point of view, these strategies could then be linked to the other five domains. They argued that specific knowledge translation strategies, such as audits, were implicit in some constructs – in this case within *goals & feedback* [8]. Powell et al. also argued that the CFIR suggests implicitly that “successful implementation may necessitate the use of an array of strategies that exert their effects at multiple levels of the implementation concept” ([7], p. 194). However, it is questionable whether such possible implementation strategies should be added as a sixth domain to the CFIR. Existing lists, such as for example the framework for implementation interventions/strategies provided by the Cochrane *Effective Practice and Organisation of Care* (*EPOC*) Review Group [13], could instead be used and its strategies mapped to the CFIR constructs.

#### Definitions of existing constructs

The CFIR’s constructs have been criticized for their wide-ranging and multi-faceted nature, with some requiring more detailed descriptions [8]. This was particularly true for the *reflecting & evaluating* construct (*process* domain), which was defined as “quantitative and qualitative feedback about the progress and quality of implementation accompanied with regular personal and team debriefing about progress and experience” [1]. This definition can be understood in two ways: as a way of continuously reflecting on and evaluating the ongoing process, but also assessing the process at the end of the implementation project from the perspective of all involved parties. This idea is in line with that of other implementation researchers [14]. In the Falls CPG implementation project in question, both perspectives were used. Assessing the implementation process from the participants’ perspective enabled relevant aspects to be captured for the implementation of further endeavours in the same setting, for example, participant learning, perceived barriers and facilitators and views/recommendations on how to ensure sustainable implementation. Ilott et al. also regarded this *reflecting & evaluating* construct as inadequate, especially in view of its importance for the achievement of longer-term change [8]. Sustainability was regarded as an additional important aspect that was lacking in the framework, but any innovation that is not implemented sustainably would be “a waste of time, financial resources and leadership effort at a time of economic austerity” ([8], p. 8).

Our results indicated that the underlying *engaging* construct (*process* domain) should not only include those persons with formal or informal power, but also all other stakeholders, because their support or opposition, expressed through often seemingly insignificant contributions, can significantly influence the implementation process. As a group, they have the power to support or hinder the innovation process. This large, but often overlooked, group of people must, therefore, be considered during the implementation process. According to Damschroder, this will be included in the second version of the CFIR [14].

Including additional underlying constructs inevitably adds to the complexity of the framework [8]. One of its potential drawbacks was the CFIR’s complexity [4]. However, if the CFIR were used for various implementation projects in one setting, not every domain and underlying construct would need to be evaluated in each new implementation project. Once an in-depth assessment had been carried out within an organisation, the existing, valid knowledge could be used to inform new projects. Not assessing each CFIR construct every time is in line with existing literature: Damschroder and Hagedorn, meanwhile, suggested evaluating the list of CFIR constructs to identify those with the highest applicability in the intended study. The assessment of the study could then focus on those relevant constructs [3]. Damschroder and Lowery concluded in a later paper that they would only include twelve constructs for guiding future implementation if they differentiated between high and low implementation effectiveness [5].

### CFIR applicability and usefulness

#### The CFIR as a guide to developing assessment questions and as a framework for revealing influential factors on the implementation process

All in all, the CFIR proved to be easily applicable and highly useful for its intended application as a guide that could be used to develop assessment questions and as a framework to reveal influential factors on the guideline implementation process. For the implementation project presented in this paper, the provided and added (sub-) constructs could be easily utilised for developing and compiling baseline, mid-term and final assessments. The majority of the constructs were clearly defined, which facilitated the development of relevant questions with respect to the search for pre-existing instruments. Our results supported the findings of Illot et al., who evaluated the CFIR as a “high-level conceptual framework that encompasses a range of concepts that are applicable to a wide variety of situations” ([8], p. 915).

#### The CFIR as a template for content-analysis and as a guide to interpret the main findings

The *CFIR*, with its added (sub-) constructs, could easily be utilised to guide qualitative data analysis within a prospective CPG implementation project. Furthermore, by applying the CFIR, information about whether the employed implementation strategies were effective and why they worked or failed was gathered. By combining all data collected during the course of the project (i.e., qualitative and quantitative data, the main investigator’s research diary records and steering group team meeting minutes) under the CFIR frame, the interrelations between the CFIR domains and constructs became visible. This helped us explain the main findings.

### Limitations

The principle limitation of this evaluation was that the assessment of how useful the CFIR actually was for guiding the implementation of a CPG during the course of implementation (i.e., the CFIR’s comprehensiveness, applicability and usefulness) was performed retrospectively. This retrospective assessment of the main investigator’s diary, steering group meeting minutes and qualitative and quantitative data were used to develop the main focus of the study as well as main investigator’s post-hoc reflections, and thus contrasts with a thoroughly and prospectively planned research study conducted with validated instruments/procedures.

## Conclusions

The CFIR proved to be a valuable and helpful, although not exhaustive, framework that could be used to assess the baseline, process and final state of a CPG implementation project. The findings of the study indicated that this framework should be supplemented with other important factors and local features to achieve a sound basis for the planning and realisation of an ongoing project. Furthermore, it was recognized that a clear definition of underlying constructs, for example *reflecting & evaluating*, would facilitate the use of the CFIR to implement an innovation. The CFIR also proved to be both applicable and useful during the development of relevant interview and group discussion questions, compilation of several data collection tools, analysis of qualitative data, and organisation of the obtained results into the CFIR’s domains and underlying constructs, which can help explain and interpret the outcomes of an implementation project.

### Relevance to clinical practice and further research

The CFIR and the authors’ proposed supplements can help nurse managers, other responsible staff and/or researchers obtain a comprehensive overview of factors influencing an implementation project. They may also facilitate the contextualisation of findings, explanation of crucial elements in the process and assessment of final outcomes of an implementation project.

This evaluation provides valuable insights that support further improvements in the general applicability and comprehensiveness of the CFIR, for example, a clear definition of constructs. The CFIR’s constructs themselves exceeded the scope of this evaluation. The validity of the CFIR constructs requires evaluation in further research projects. The results of the retrospective evaluation of the CFIR in question should be confirmed and refined as part of a carefully planned, prospective research project in the future.

## Additional File

Additional file 1:
**Data collection tools and data analysis.** Description of questionnaires and semi-structured interview guides as well as mode of data analysis. (DOC 118 kb)

## Abbreviations

ASD: Accident Surgery Department
CFIR: Consolidated Framework for Implementation Research
CPGs: Clinical practice guidelines
i.e.: That is to say
OD: Ophthalmic Department
PAR: Participatory action research
RD: Research diary
SGMM: Steering-group-meeting minutes
t1: Baseline data collection
t2: Mid-term data collection
t3: Final data collection
