# Supplementary material for: The Consolidated Framework for Implementation Research (CFIR): a useful theoretical framework for guiding and evaluating a guideline implementation process in a hospital-based nursing practice
Source: BMC Nurs. 2015 Aug 12;14:43. doi: 10.1186/s12912-015-0088-4 (PMC4533946; doi:10.1186/s12912-015-0088-4)
Supplement: Additional file 1: — Data collection tools and data analysis. Description of questionnaires and semi-structured interview guides as well as mode of data analysis. (DOC 118 kb) [file 12912_2015_88_MOESM1_ESM.doc]

**Additional File 1: Data collection tools and data analysis**

Breimaier HE, Halfens RJG, Heckemann B, Lohrmann C: **The Consolidated Framework for Implementation Research (CFIR): a useful theoretical framework for guiding and evaluating a guideline implementation process in a hospital-based nursing practice**

**Data collection tools**

The Consolidated Framework for Implementation Research (CFIR) [1] was used to determine the focus for each data collection point. During the baseline (t1) period, the aim was to assess factors that influenced the intervention (guideline implementation), placing an emphasis on the intervention characteristics (e.g., familiarity with the Falls CPG), inner and outer settings (e.g., culture of the organisation, peer pressure) as well as characteristics of the individuals such as demographics. The *process* domain, with a focus placed on the evaluation of the implementation process from the participants’ perspective, was introduced during the mid-term (t2) period and was the main focus for the final data collection (t3). Data were collected via questionnaire, during group discussions and semi-structured interviews. The questionnaires (t1 & t2) included 13 closed-ended questions about personal attributes, two closed-ended questions about the Falls CPG and five instruments to assess participant attitudes towards guidelines, fall preventive knowledge, organisational culture and organisational learning capability. The questionnaire that was used for the final data collection additionally included six instruments that focussed on several aspects of the implementation process and its consequences. More details are presented in Table 1, in which these quantitative measures are also mapped against the CFIR.

**Table 1 – Quantitative measures mapped against the Consolidated Framework for Implementation Research (CFIR) domains**

| **CFIR domains** | **Quantitative measures used for assessing influential factors within the Falls CPG implementation process** |
| --- | --- |
| **Characteristics of the individuals*** | *Closed-ended demographics questions:* age, gender, profession, department |
|  | *Other personal attributes:* fall incidence experienced during nursing career; work experience in current position; part-time employment; year of diploma; participation in further training (nursing science, nursing research, evidence-based nursing); inclusion of research-relevant aspects in nursing education; how often research articles are read; and participation in baseline and/or mid-term data collection |
|  | *General Self-Efficacy Scale:* measuring participants’ self-efficacy. The scale features 10 items on a 4-point Likert scale from 1 = *not at all true* to 4 = *exactly true* [2]. The individual response scores were totalled and divided by ten. The final composite score ranged from 1.0 to 4.0. A higher score reflected a person’s more optimistic outlook. The scale was accessible for free in German [3]. |
|  | *Nursing personnel’s knowledge*: 13 items (7 single- and 6 multiple-choice items, 81 answer options), developed for this study, measured nursing personnel knowledge about the guideline in terms of risk of falls, fall prevention and recommended measures. Further information is provided in Breimaier et al. [4]. |
|  | *Attitudes Towards Guidelines Scale:* used to measure nursing personnel’s attitudes towards guidelines. The scale consisted of seven subscales: *general attitude, usefulness, reliability, lack of individual or team competence, lack of organisational competence, impracticality* and *availability.* Each subscale consisted of two Likert-scaled items from 1 = *strongly disagree* to 4 = *strongly agree* [5]. The translation process was described in Breimaier et al. [4]. |
| **Inner setting*** | *Competing Values Framework (CVF):* The organisational culture of the two participating departments was measured using this 20-item instrument [6] with a 4-point Likert scale (1 = *does not apply;* 4 = *applies*). Four different, but equivalent, organisational cultures could be distinguished [6, 7]:   1. *group culture:* the values and norms emphasised are associated with affiliation, teamwork and participation 2. *developmental culture:* characterised by the promotion of risk taking in conjunction with innovation and change 3. *hierarchical culture:* emphasis on stability, rules, policies and regulations; it reflects the norms and values associated with bureaucracy 4. *rational culture:* emphasis on efficiency and achievement   Each culture was composed of five statements regarding *group character, leadership style, cohesion, strategic emphasis* and *rewards of a group* [8]. Permission to use this instrument was obtained from the developer. The translation proceeded by following five steps described in Beaton et al. [9], but only one native English translator was used. |
|  | *Organisational Learning Survey (OLS) Instrument:* The organisational learning capability of the two departments was measured using 21 items on a 5-point Likert scale (from 1 = *strongly disagree* to 5 = *strongly agree*) [10]. The five key conditions measured were defined according to Goh and Richards as the following ([10], p. 578):   1. *Clarity of purpose and mission* (4 items): the degree to which nursing personnel have a clear vision/mission of the organisation and understand how they can contribute to its success and achievement. 2. *Leadership commitment and empowerment* (5 items): the role of the nursing director, head and ward managers in the organisation with respect to helping nursing personnel learn and elicit behaviours that are consistent with an experimenting and changing culture. 3. *Experimentation and rewards* (5 items): the degree of freedom nursing personnel enjoy in exploring new ways of getting the job done and expressing the freedom to take risks. 4. *Transfer of knowledge* (4 items): the systems that enable nursing personnel to learn from others, past failures and other organisations. 5. *Teamwork and group problem solving* (3 items): the degree of interdisciplinary teamwork used in the organisation to solve problems and generate new and innovative ideas.   The developers gave their permission to use this instrument, and the translation followed the same procedure described above. |
| **Process evaluation**** | The guideline implementation process was evaluated using an instrument developed by Caldwell et al [11]. Its six parts were: *characteristics of the change process* (10 items); *extent of work unit change* (3 items); *consequence of guideline implementation* (4 items); *individual job impact* (6 items); *demands-abilities (person-job) fit* and *values-congruence (person-organisation) fit* (each 2 items) [11]. The Likert scale ranged from 1 = *strongly disagree* to 5 = *strongly agree*. Caldwell gave permission to use and adapt the instrument to the nursing context. The translation process followed the procedure described above. |
|  | An additional question was asked: whether the educational meetings had been supportive in implementing the Falls CPG. The Likert scale ranged from 1 = *strongly disagree* to 5 = *strongly agree*. |

* Asked at all three data collection time points (t1 – t3); ** Asked at the final data collection time point (t3)

Interview guides for the semi-structured interviews and discussions were based on the CFIR framework and featured open-ended questions that pertained to each respective implementation stage. Table 2 presents the CFIR domains focussed upon at each data collection time point with respective examples. In order to gain a second opinion from a nursing expert familiar with qualitative research, BH reviewed and discussed the content of the interview guides (t2 and t3) with HEB with regard to relevance. The final questions were determined by consensus. All discussions and interviews were audiotaped and verbally transcribed either by the primary investigator or a research assistant. All transcripts were reviewed for accuracy and proofread by HEB.

**Table 2 – Topics of interview guidelines mapped against the Consolidated Framework for Implementation Research (CFIR) domains**

| **Data collection point** | **Foci of semi-structured interview guides with examples** |
| --- | --- |
| **Baseline (t1)** | *Characteristics of intervention*  Participant’s perception of the issue, familiarity with the Falls CPG content, anticipated benefits |
|  | *Inner setting*  Local workflow and communication channels, workload, teamwork, tension for change |
|  | *Characteristics of the individuals*  Participants’ expectations about the implementation of the Falls CPG |
|  | *Additional relevant information*   - Stakeholders’ aims when implementing the Falls CPG - Stakeholders’ wishes/needs about the implementation of the Falls CPG - Pre-established measures for preventing falls - Pre-established strategies for implementing an innovation |
| **Mid-term (t2)** | *Characteristics of the intervention*  Applicability and fit of the content/measures introduced, perceived time investment into the implementation process |
|  | *Inner setting*  Transparency of the process; participant satisfaction with goals already achieved, opportunity to express own ideas and/or criticisms |
|  | *Characteristics of individuals*  Knowledge related to and attitude towards the Falls CPG, change in own mode of work |
|  | *Process*  Interviewee satisfaction with the progress of the implementation; its impact on daily work; perceived difficulties; perceived benefits; perceived changes; recommendations for further implementation projects |
| **Final data collection (t3)** | *Characteristics of intervention*  Nursing personnel’s perception about source and quality of the Falls CPG; to what degree the Falls CPG could be adapted, applicable and useful; familiarity with the Falls CPG; perceived time investment into the implementation process |
|  | *Inner setting*  Access to the Falls CPG and materials compiled during steering group meetings |
|  | *Characteristics of individuals*  Knowledge related to and attitude about the Falls CPG |
|  | *Process*  Participant’s thoughts on the implementation process; perceived difficulties and changes; meeting of expectations; satisfaction with the implementation strategies, perceived changes |

During the baseline (t1) period, general information about the features of the setting (*inner setting* domain, *structural characteristics* construct) were obtained from the head nurse including the number of beds, employed nursing personnel, staff turnover, patient length of stay and patients’ conditions. To evaluate the ongoing implementation process within the steering group meetings, the discussions were recorded digitally and summarized in the respective protocol.

**Data analysis**

Quantitative data were analysed descriptively (mean, standard deviation, percentages, frequency count) using PASW Statistics for Windows, Version 18 (t1) / IBM SPSS Statistics for Windows, Version 20 (t3).

Qualitative data were content-analysed as described below and with MAXQDA 10. The CFIR, supplemented with the four constructs *stakeholders’ aims* and *stakeholders’ wishes/needs when implementing an innovation*, *pre*-*established fall prevention measures* and *pre*-*established strategies for implementing an innovation*, provided a template for the analysis. The unit of analysis was made up of the two participating departments, and the meaning units were sentences that contained related aspects with regard to content and context [12]. In the first step, the manifest content [12] of each transcript was categorised using the CFIR, and the four supplemental (sub-) constructs and their content were summarised as a memo. If additional interview data were found by the researchers to be relevant for the implementation process, but did not fit into the already existing (sub-) constructs, additional (sub-) constructs were added over the course of the analysis process such as *participants’ involvement*. Secondly, the memos from all interviews and all discussions at the data collection time point were scrutinised for commonalities, and were subsequently labelled and summarized. The content that was categorised into the *learning effect* construct (*process* domain) revealed, for example, that participants perceived learning effects with regard to the comprehensibility of the Falls CPG; further devices to prevent falls; the change process, and insights into nursing science and research. To strengthen the credibility of data categorisation, member checks were done after the baseline data were analysed. Participants confirmed that their views were reflected in the proposed constructs. Mid-term and final data categorisation (step 1 and step 2) were counterchecked by BH. Discrepancies were discussed between HEB and BH until an agreement was reached [11].

**References**

1. Damschroder LJ, Aron DC, Keith RE, Kirsh SR, Alexander JA, Lowery JC. Fostering implementation of health services research findings into practice: a consolidated framework for advancing implementation science. *Implement Sci.* 2009*;*4:50. doi: 10.1186/1748-5908-4-50.
2. Jerusalem M, Schwarzer R. Allgemeine Selbstwirksamkeitserwartung SWE [userpage.fu-berlin.de/health/germscal.htm].
3. Schwarzer R, Jerusalem M. The General Self-Efficacy Scale (GSE) [http://userpage.fu-berlin.de/~health/engscal.htm].
4. Breimaier HE, Halfens RJG, Lohrmann C. Effectiveness of multifaceted and tailored strategies to implement a fall-prevention guideline into acute care nursing practice: a before-and-after, mixed-method study using a participatory action research approach. *BMC Nurs.* 2015;14:18. doi: 10.1186/s12912-015-0064-z.
5. Elovainio M, Eccles M, Mäkelä M. Attitudes towards guidelines and a scale for measuring them. In Thorsen T, Mäkelä M, editors. *Changing professional practice. Theory and practice of clinical guidelines implementation.* Copenhagen: Danish Institute for Health Services Research and Development; 1999. p. 153-67.
6. Shortell SM, Zazzali JL, Burns LR, Alexander JA, Gillies R, Budetti PP, Waters TM, Zuckerman HS. Implementing evidence-based medicine. The role of market pressures, compensation incentives, and culture in physician organizations. *Med Care.* 2001;39:I-62-78.
7. Shortell SM. Quality improvement implementation survey II. University of California, Berkeley; 1992. [http://shortellresearch.berkeley.edu/CABG_THR/QUALIMP9_1queALL_ssc.doc].
8. Shortell SM, Marsteller JA, Lin M, Pearson ML, Wu S-Y, Mendel P, Cretin S, Rosen M. The role of perceived team effectiveness in improving chronic illness care. *Med Care*. 2004;42:1040-48.
9. Beaton DE, Bombardier C, Guillemin F, Bosi Ferraz M. Guidelines for the process of cross-cultural adaptation of self-report measures. *SPINE.* 2000;25:3186-91.
10. Goh S, Richards G. Benchmarking the learning capability of organizations. *Eur Manag J.* 1997;15:575-83.
11. Caldwell SD, Herold DM, Fedor DB. Toward an understanding of the relationships among organizational change, individual differences, and changes in person-environment fit: a cross-level study. *J Appl Psychol.* 2004;89:868-82.
12. Graneheim UH, Lundman B. Qualitative content analysis in nursing research: concepts, procedures and measures to achieve trustworthiness. *Nurse Educ Today.* 2004;24:105-12.
